# Evidence of the validity of a novel version of the computerized cognitive screening battery CompCog

**DOI:** 10.1590/1980-57642021dn15-040010

**Published:** 2021

**Authors:** Larissa Hartle, Liana Mendes-Santos, Eduarda Barbosa, Giulia Balboni, Helenice Charchat-Fichman

**Affiliations:** 1 Department of Psychology, Pontifícia Universidade Católica do Rio de Janeiro – Rio de Janeiro, RJ, Brazil.; 2 Department of Philosophy, Social, Human and Education Sciences, Università degli Studi di Perugia – Perugia, Italy

**Keywords:** reaction time, cognitive assessment screening instrument, neuropsychological tests, cognition, tempo de reação, instrumento de triagem de avaliação cognitiva, testes neuropsicológicos, cognição

## Abstract

Although the availability of the computer-based assessment has increased over the years, neuropsychology has not carried out a significant paradigm shift since the personal computer’s popularization in the 1980s. To keep up with the technological advances of healthcare and neuroscience in general, more efforts must be made in the field of clinical neuropsychology to develop and validate new and more technology-based instruments, especially considering new variables and paradigms when compared to paper and pencil tests.

**Objective:**

This study’s objective was to produce concurrent validity evidence of the novel version of the computerized cognitive screening battery CompCog.

**Methods:**

Participants performed a traditional paper and pencil neuropsychological testing session and another session where CompCog was administrated. The data of a total of 50 young adult college students were used in the analyses.

**Results:**

Results have shown moderate and strong correlations between CompCog’s tasks and their equivalents considering paper and pencil tests. Items clustered in agreement with the subtest division in a principal component analysis.

**Conclusions:**

The findings suggest that CompCog is valid for measuring the cognitive processes its tasks intend to evaluate.

## RESUMO

Embora a disponibilidade de instrumentos computadorizados para avaliação tenha aumentado ao longo dos anos, a neuropsicologia não passou por uma mudança significativa de paradigma desde a popularização do computador pessoal nos anos 1980. Para acompanhar os avanços tecnológicos da saúde e da neurociência em geral, mais esforços devem ser feitos no campo da neuropsicologia clínica para desenvolver e validar novos instrumentos de base mais tecnológica, especialmente considerando novas variáveis e paradigmas quando comparados aos testes de lápis e papel.

**Objetivo:**

O objetivo deste estudo foi produzir evidências de validade concorrente da nova versão da bateria computadorizada de rastreio cognitivo CompCog.

**Métodos:**

Os participantes passaram por uma sessão de avaliação neuropsicológica com testes tradicionais de lápis e papel e de outra sessão em que o CompCog foi administrado. Os dados do total de 50 jovens adultos universitários foram utilizados nas análises.

**Resultados:**

Os resultados mostraram correlações moderadas e fortes entre as tarefas do CompCog e seus equivalentes nos testes tradicionais. Uma análise de componentes principais mostrou que os itens formaram fatores em concordância com a divisão de subtestes da bateria.

**Conclusões:**

Os resultados sugerem que o CompCog é válido para medir os processos cognitivos que suas tarefas pretendem avaliar.

## INTRODUCTION

New technologies are powerful tools to aid in diagnosis and health treatments. In the clinical neuropsychological context, technology can be incorporated into the cognitive evaluation, stimulation, training, and rehabilitation. However, since the personal computer’s popularization in the 1980s, neuropsychology has not gone through a significant paradigm shift.^1^ The availability of the computer-based assessment has increased over the years, especially over the past decade,^2^ but the neuropsychological testing still relies primarily on paper and pencil tasks.^3^ The use of computerized tests is still rare,^1^ and many of the efforts made to keep up with the technological advancements are related to the development of a computerized version of a traditional paper and pencil test.^4^ A meta-analysis found that they are usually equivalent.^5^ Also, there is a criticism that both are based on the theoretical concepts developed decades ago, resulting in a “cosmetic change” only.^3^

Some effort has also been made in the software development related to the scoring of those traditional tests.^1^ Thus, in general, the studies are divided into two groups: (1) the adaptation of the existing standardized tests to the computer and (2) the development of new tests and batteries for evaluating cognitive functions, which range from the design and build to the validation of new computerized instruments. The examples of some traditional and widely used neuropsychological tests that were adapted to computerized formats are the Wisconsin Card Sorting Test (WCST),^4^ the Category Test — a section of the Halstead-Reitan Neuropsychological Test Battery,^6,7^ versions of the Raven’s Progressive Matrices,^8^ and the Corsi Block task.^9^

The examples among the computerized batteries that were developed for the evaluation of cognitive functions are the Automated Neuropsychological Assessment Metrics (ANAM), which evaluates attention, concentration, reaction speed, memory, mathematical ability, and decision-making;^10^ the Cambridge Neuropsychological Test Automated Battery (CANTAB), which evaluates working memory and planning, visuospatial memory, and attention;^11^ the Central Nervous System (CNS) Vital Signs (CNVS), which evaluates memory, cognitive flexibility, psychomotor speed, time, reaction, and complex attention;^12^ the Computerized Neuropsychological Test Battery (CNTB), which evaluates information processing, motor speed, verbal and spatial memory, attention, language, and spatial abilities;^13^ the Mindstreams, which evaluates memory, executive functions, attention visuospatial, verbal fluency, motor skills, and information processing;^14^ the Cognition assessment using the NIH Toolbox, which evaluates executive function, episodic memory, language, processing speed, working memory, and attention;^15^ and the Computerized Neurocognitive Scanning, which evaluates executive function, memory, intellectual, and sensorimotor functions.^16^

But to keep up with the technological advances of healthcare and cognitive neuroscience, in general, more efforts must be made in the field of clinical neuropsychology to develop and validate new and more technology-based instruments considering variables known to be valuable and that technology made it possible to use such as processing speed and reaction time.^17^ The paper and pencil neuropsychological tests hardly have precise reaction time measurements, but this is an advantage that stands out among computerized tests: specific and complex variables, such as reaction time, can be measured even in milliseconds.^18^ Reaction time is a variable that seems to be affected throughout many types of neurodegenerative conditions,^19-22^ and the present computerized battery focuses on the reaction time as its primary measure — although it is also able to measure errors. Also, the reaction time is measured as “median reaction time” throughout all the tests, so some concerns considering variability, which have been raised, could be addressed.^23^ Other computerized batteries using reaction time variables already exist and presented good evidence validity.^24^ Still, they are available only in English and use a mean reaction time, instead of a median. In this context, this study’s objective was to produce concurrent validity evidence of the novel version of the computerized cognitive screening battery CompCog. CompCog was initially called as Bateria de Testes Neuropsicológicos Computadorizados^25^ (BTNC; Brazilian Portuguese version). It was created using the MEL Professional version 2.0^26^ to evaluate anterograde episodic memory, attention, visual perception, processing time information, and short-term memory. The first study concerning it investigated the clinical markers of early Alzheimer’s disease (AD). Forty individuals with mild AD and 73 controls, paired for age and education, were studied. The battery had six tests, and the application lasted 40 min on average. It was run on an IBM-PC-compatible microcomputer using a 14-inch SVGA color monitor. A keypad with five buttons, labeled from 1 to 5, was used as a response input device. The AD group showed a significantly lower percentage of a correct response on episodic memory and short-term memory and a higher latency response on all other tests when compared to controls. The receiver operating characteristic (ROC) analysis showed that episodic memory, short-term memory, and choice reaction time tests were sensitive and specific to discriminate the groups and, therefore, were the clinical markers of early AD.^18^

After that study, a screening version of the instrument was created. It took only 15 min to be administrated and was named Computerized Cognitive Screening test — CompCogs. CompCogs had the same material as BTNC, was developed with MEL Professional, was run on an IBM-PC, and had the same keypad with five buttons as a response input device. The CompCogs was applied in 47 individuals with probable mild AD and 97 controls. The idea was to investigate its validity for the early diagnosis of AD. CompCogs presented 91.8% sensitivity and 93.6% specificity for AD diagnosis using the ROC analysis of AD diagnosis probability derived by logistic regression. It showed high validity for AD early diagnosis and, therefore, may be a useful alternative screening instrument.^27^

In 2011, CompCog was developed for mobile devices that operate on the iOS operating system, maintaining the original ideas of evaluation, but now with a new way of interaction — the touchscreen, more dynamic interface, and the possibility to be carried out in the iPad. This new version is broad and flexible. Although there is a suggested order of application, the examiner can select the tests and their order. It comprises eight tests that evaluate different cognitive domains such as information processing speed and reaction time, implicit, episodic, working memory, attention, and inhibitory control. Battery administration lasts about 40 min in healthy individuals.

The test is available in Portuguese, Spanish, and English. The demographical data is collected at the beginning, such as full name, age, education, sex, and handiness. The results of each test are presented at the end of each application and at the end of the whole battery. All data are stored in the cloud and are available in an Excel spreadsheet accessible through the test’s website. All answers are issued using a touch screen and recorded. All tests generate reaction time measures registered in milliseconds for each touch, both as total time and as a median, to eliminate the eventual discrepant data through each test. Furthermore, correct responses’ percentage, errors, and differences in reaction time between errors and correct answers are also registered. All stimuli tests are visuospatial, except for one test — Stroop test, which contains written words in order to maintain the original paradigm.

## METHODS

### Setting and participants

The study took place at the Pontifical Catholic University of Rio de Janeiro, where the undergraduate psychology-level students were recruited and they performed the data collection at the university’s psychology clinic. Participants performed a traditional paper and pencil neuropsychological testing session and another session where CompCog was administrated.

Initially, 64 young adults were selected. However, 14 were excluded: 10 for psychiatric disorders, 1 for a neurological disorder, 2 for metabolic disorders, and 1 for drug use. Thus, the data of a total of 50 participants were used in the analyses of concurrent validity evidence. The mean age in years was 21.18 (4.02), and they had a mean of 13.5 (1.7) years of schooling, being 86% women.

### Materials

#### CompCog

This research used the test’s standard tasks’ order in Portuguese during the data collection phase. Tests are explained, with their variables and cognitive functions assessed, in Table 1.

**Table 1.** CompCog test’s variables.

Test	Cognitive functions involved and how they are evaluated	Variables
Simple Reaction Time	Processing speed. As soon as a white square appears in the middle of the screen, the person should touch the rectangle in the bottom of the screen.	Median reaction time, total time
Choice Reaction Time	Processing speed. As a white or orange square appears in the middle of the screen, the person should touch the rectangle of the same color in the bottom of the screen.	Median reaction time, total time, correct answers
Implicit Learning Test	Implicit learning. As 1 of 10 gray squares distributed in the screen turns white, the person must press it. There is a fixed sequence of 25 squares that is repeated four times (sequence 1–4) and one last random sequence for control (sequence 5).	Implicit learning (median reaction time in sequence 4 – median reaction time in sequence 1) and implicit learning interference (median reaction time in sequence 5 – median reaction time in sequence 1)
Visual and Spatial Short-Term Memory	Working memory. There are 10 gray squares distributed on the screen. One will become white at a time, making a sequence that must be reproduced.	Correct answers, direct order SPAN, reaction time in direct order, inverse order SPAN, reaction time in direct order
Face Recognition and Memory	Episodic memory. A total of 10 drawings of unknown faces are presented for 30 s. Then, the participant should choose, between 10 pairs of faces, which one was among those initially shown for memorization in 4 attempts.	Total time and correct answers, and median reaction time and correct answers for each of the four tasks.
Inhibitory Control Test	Attention, Inhibitory control Squares of different colors will appear in the middle of the screen for 1 s each, the white ones should be avoided.	Total time, median reaction time, correct answer median reaction time, error’s median reaction time and errors
Stroop Test	Attention, Inhibitory control. All tasks have four colored rectangles located at the bottom of the screen. The person should touch the one matching the stimuli that appear in the middle of the screen considering its color without (task 1) and with distracters (tasks 2 and 3).	Total time, interference, median reaction time and errors for each of the three tasks
Survey Test	Attention. Squares of different colors will appear in the middle of the screen for 1 s each. Participants should press the white ones on the first tasks, white and blue ones on the second, and yellow ones on the third.	Median reaction time and correct answers for each of the three tasks

#### Paper and pencil tests

The neuropsychological assessment was performed through a paper and pencil battery that included traditional tests commonly used in clinical neuropsychology in Brazil. Tests used and their variables are shown in Table 2.

**Table 2.** Traditional test’s variables.

Test	Cognitive functions involved	Variables
TEACO-FF	Attention, Processing speed	Total score
TECON 1	Attention, Processing speed	Total score
Stroop Test	Attention, Inhibitory control	Errors and time to completion in each task, interference
Color Trails Test	Attention, Inhibitory control	Time to completion in each task, interference
WAIS-III processing speed index	Attention, Processing speed	Coding, Symbol Search, Processing Speed Index
WAIS-III working memory index	Working memory	Arithmetic, Digit SPAN and Letter-Number Sequencing, Working Memory Index, direct order SPAN, inverse order SPAN
Rey Auditory Verbal Learning Test	Episodic memory	A1, A2, A3, A4, A5, A6, A7, B1, proactive interference, retroactive interference, forgetful speed, recognition
Rey–Osterrieth Complex Figure Test	Episodic memory	Total score and time to completion in copy and recall
Wisconsin Card Sorting Test	Implicit learning	Number of categories completed, trials to complete first category, failure to maintain set, learning to learn
R-1 Test	Implicit learning	Total score

#### Equivalence

The cognitive measures assessed through the traditional tests were compared to the ones evaluated by CompCog tasks, as shown in Table 3.

**Table 3.** Cognitive functions measured by traditional tests and predicted equivalents.

Cognitive function	Traditional tests	CompCog tests
Attention	TEACO-FF, TECON 1, Stroop Test, Color Trails Test, WAIS-III processing speed index	Inhibitory Control Test, Stroop Test, Survey Test
Working memory	WAIS-III working memory index (Arithmetic, Digit Span, and Letter-Number Sequencing).	Visual And Spatial Short-Term Memory
Episodic memory	Rey Auditory Verbal Learning Test, Rey–Osterrieth Complex Figure Test	Face Recognizing And Memory
Implicit learning	Wisconsin Card Sorting Test, R-1 Test	Implicit Learning Test
Inhibitory control	Stroop Test, Color Trails Test	Inhibitory Control Test, Stroop Test
Processing speed	TEACO-FF, TECON 1, WAIS-III processing speed index (Digit Symbol-Coding and Symbol Search)	Simple Reaction Time, Choice Reaction Time

#### Ethics

This study was registered at the university’s Ethics Research Committee and authorized by the 2012–31 Favorable Opinion. The volunteers participated in the study by signing a free and informed consent form, according to resolution 196/96 of Brazil’s National Health Council, which deals with the guidelines and standards for research involving human subjects. Participation in this survey was voluntary, so they did not receive any payment. The study did not bring any risk to volunteers’ health, and they could refuse and/or withdraw consent to participate in the study at any time.

#### Statistical methods

All data entry and analysis were carried out using SPSS Windows 22.0. Pearson’s correlations were run between traditional paper and pencil variables and CompCog variables. The p-values under 0.05 were considered to show statistical significance. A principal component analysis (PCA) with oblimin rotation (delta=0) was conducted on all items regarding time measures. ­Components were extracted based on eigenvalues of more than 1. Since the size sample was small, we used the Kaiser-Meyer-Olkin (KMO) measure to verify the sampling adequacy for the analysis. KMO was 0.662, above the level of 0.5 for adequacy. Bartlett’s test of sphericity (χ^2^ (300)=1658.970, p<0.001) indicated that correlations between items were sufficiently large for PCA.

## RESULTS

Results have shown moderate and strong correlations between CompCog’s tasks and their equivalents considering paper and pencil tests. The results of the correlations are shown in Table 4, regarding comparisons reported in Table 1 with variables reported in Tables 2 and 3. ­Participant’s performance in each test are reported in Table 5. Regarding the PCA, Table 6 shows the pattern matrix, with extracted components’ loadings (i.e., regression coefficients) after oblimin rotation. The Phi matrix is reported in Table 7. In general, each subtest’s items — except for items of Simple Reaction Time and Choice Reaction Time, which clustered together – loaded on the same component, generating seven components in total.

**Table 4.** Correlations between cognitive measures assessed through the traditional tests and CompCog.

	TEACO-FF	TECON 1	Processing Speed Index	WCST: categories completed	WCST: trials to complete first category	WCST: failure to maintain set	WCST: learning to learn	R-1	Working Memory Index	Direct order SPAN	Inverse order SPAN
Simple reaction time and choice reaction time tests											
Median simple reaction time	-0.289, p=0.042*	-0.510, p<0.001***	-0.196, p=0.171								
Total simple reaction time	-0.330, p=0.019*	-0.546, p<0.001***	-0.245, p=0.086								
Median choice reaction time	-0.524, p<0.001***	-0.503, p<0.001***	-0.478, p<0.001***								
Total choice reaction time	-0.562, p<0.001***	-0.489, p<0.001***	-0.508, p<0.001***								
Correct answers	-0.101, p=0.485	-0.243, p=0.089	-0.052, p=0.722								
Implicit learning test											
Implicit learning				0.073, p=0.614	-0.156, p=0.279	-0.214, p=0.135	-0.198, p=0.167	-0.305, p=0.031*			
Implicit learning interference				0.094, p=0.515	-0.410, p=0.003**	-0.081, p=0.575	-0.131, p=0.365	0.125, p=0.387			
Visual and spatial short-term memory test											
Correct answers									-0.357, p=0.011*	-0.288, p=0.042*	0.224, p=0.118
Direct order SPAN									0.339, p=0.016*	0.173, p=0.229	0.224, p=0.118
Reaction time in direct order									-0.121, p=0.404	-0.186, p=0.195	-0.077, p=0.594
Inverse order SPAN									0.100, p=0.491	0.149, p=0.301	-0.205, p=0.153
Reaction time in direct order									-0.134, p=0.353	-0.179, p=0.213	-0.064, p=0.658
Survey test											
Median reaction time 1st task	-0.479, p<0.001***	-0.274, p=0.054									
Correct answers 1st task	0.138, p=0.339	0.226, p=0.114									
Median reaction time 2nd task	-0.512, p<0.001***	-0.171, p=0.263									
Correct answers 2nd task	0.119, p=0.411	-0.173, p=0.229									
Median reaction time 3rd task	-0.495, p<0.001***	-0.083, p=0.556									
Correct answers 3rd task	0.016, p=0.910	-0.061, p=0.674									

	RAVLT A3	RAVLT A4	RAVLT A5	RAVLT A1–A5	RAVLT A6	RAVLT A7	RAVLT recognition	Rey Figure Recall score	Rey Figure Recall time	Stroop test 1st task	Stroop test 2nd task	Stroop test 3rd task	Stroop test Interference	Color Trails 2nd task
Face recognition and memory test														
Correct answers	0.342, p=0.015*	0.345, p=0.014*	0.309 p=0.029*	0.384, p=0.006**	0.328, p=0.020*	0.110, p=0.446	0.332, p=0.018*							
Correct answers task 1	0.345, p=0.014*	0.363, p=0.010**	0.207, p=0.148	0.339, p=0.016*	0.302 p=0.033*	0.111, p=0.443	0.259, p=0.070							
Correct answers task 2	0.222, p=0.122	0.191, p=0.185	0.099, p=0.496	0.156, p=0.279	0.251, p=0.079	0.147, p=0.307	0.146, p=0.311							
Correct answers task 3	0.142, p=0.326	0.104, p=0.473	0.323, p=0.022*	0.203, p=0.157	0.251, p=0.079	0.147, p=0.307	0.274, p=0.054							
Correct answers task 4	0.025, p=0.865	0.141, p=0.329	0.141, p=0.329	0.122, p=0.399	0.016, p=0.912	0.062, p=0.670	0.117, p=0.417							
Total time								-0.305, p=0.031*	0.166, p=0.250					
Median reaction time task 1								-0.378, p=0.007**	0.177, p=0.219					
Median reaction time task 2								-0.309, p=0.029*	-0.065, p=0.653					
Median reaction time task 3								-0.156, p=0.281	0.041, p=0.778					
Median reaction time task 4								-0.190, p=0.186	-0.044, p=0.760					
Inhibitory control test														
Total time												0.341, p=0.015*		0.055, p=0.702
Median reaction time												0.358, p=0.011*		0.086, p=0.550
Correct answer median reaction time												0.358, p=0.011*		0.082, p=0.573
Errors median reaction time												0.193, p=0.180		0.374, p=0.008**
Errors												-0.175, p=0.225		-0.164, p=0.226
Stroop test														
Total time										0.050, p=0.732	0.353, p=0.012*	0.418, p=0.002**	0.331, p=0.019*	
Median reaction time in 1st task										0.137, p=0.343	0.382, p=0.006**	0.235, p=0.101	0.108, p=0.457	
Median reaction time in 2st task										0.096, p=0.509	0.403, p=0.004**	0.336, p=0.009**	0.250, p=0.079	
Median reaction time in 3st task										0.071, p=0.623	0.321, p=0.023*	0.427, p=0.002**	-0.316, p=0.025*	
Interference										0.009, p=0.952	0.154, p=0.284	0.360, p=0.010	0.301, p=0.034*	

WCST: Wisconsin Card Sorting test.

*** p<0.001;

** p<0.01;

* p<0.05.

RAVLT: Rey Auditory Verbal Learning Test.

*** p<0.001;

** p<0.01;

* p<0.05.

**Table 5.** Participants’ performance in each test.

	M (SD)	Minimum	Maximum
Face recognition and memory test			
Correct answers	99.550 (1.400)	92.5	100.0
Correct answers task 1	98.800 (4.798)	70.0	100.0
Correct answers task 2	100.0 (000)	100..0	100.0
Correct answers task 3	99.800 (1.414)	90.0	100.0
Correct answers task 4	99.600 (1.979)	90.0	100.0
Total time	49.518 (6.452)	37.430	72.544
Median reaction time task 1	1393.089 (230.010)	981.453	2214.585
Median reaction time task 2	1149.122 (146.242)	874.565	1687.089
Median reaction time task 3	1093.336 (144.033)	825.360	1495.029
Median reaction time task 4	1032.570 (135.619)	855.314	1567.207
Inhibitory control test			
Total time	62.038 (4.760)	49.838	74.377
Median reaction time	515.917 (54.208)	424.277	650.189
Correct answer median reaction time	517.0917 (53.719)	425.139	650.189
Errors median reaction time	421.378 (330.271)	000.000	1001.523
Errors	1.760 (1.697)	0.0	8.0
Stroop test			
Total time	239.458 (25.960)	202.829	336.983
Median reaction time in 1st task	696.272 (44.247)	629.101	786.947
Median reaction time in 2st task	767.973 (71.369)	646.200	1013.842
Median reaction time in 3st task	808.564 (104.696)	662.537	1197.787
Interference	1.161 (0.133)	0.994	1.690
Simple and choice reaction time tests			
Median simple reaction time	345.527 (81.091)	217.987	554.009
Total simple reaction time	36.192 (8.694)	25.36	60.34
Median choice reaction time	525.327 (60.003)	434.597	737.471
Total choice reaction time	53.736 (6.317)	43.99	74.77
Correct answers	96.800 (2.329)	91.0	100.0
Implicit learning test			
Implicit learning	-61.292 (46.071)	-192.904	15.166
Implicit learning interference	-3.641 (26.631)	-96.040	32.97
Visual and spatial short-term memory test			
Correct answers	78.260 (6.075)	65.26	90.24
Correct answers 3rd task	96.040 (3.294)	88.0	100.0
Direct order SPAN	5.700 (1.182)	3.0	8.0
Reaction time in direct order	519.482 (128.787)	271.614	871.155
Inverse order SPAN	4.680 (1.168)	1.0	7.0
Reaction time in direct order	522.952 (111.046)	335.266	878.779
Survey test			
Median reaction time 1st task	544.128 (61.712)	438.921	681.443
Correct answers 1st task	99.360 (1.481)	94.0	100.0
Median reaction time 2nd task	535.730 (48.314)	458.359	667.748
Correct answers 2nd task	99.120 (1.814)	92.0	100.0
Median reaction time 3rd task	528.489 (47.660)	424.811	633.226
Correct answers 3nd task	96.040 (3.294)	88.0	100.0

M: mean; SD: standard deviation.

**Table 6.** Pattern matrix.

	1	2	3	4	5	6	7
Face recognition and memory test							
Total time	0.089	**0.937**	0.063	-0.004	0.002	0.044	0.081
Median reaction time task 1	-0.081	**0.899**	-0.006	-0.062	-0.083	0.098	0.170
Median reaction time task 2	-0.056	**0.781**	0.065	-0.074	0.203	0.152	-0.229
Median reaction time task 3	0.118	**0.693**	0.280	0.053	0.161	0.017	-0.141
Median reaction time task 4	0.140	**0.676**	-0.071	-0.017	0.140	-0.343	0.050
Inhibitory control test							
Total time	-0.110	0.115	**0.952**	-0.060	-0.074	0.083	0.062
Median reaction time	0.007	0.041	**0.955**	-0.076	0.000	-0.078	-0.059
Correct answer median reaction time	0.013	0.057	**0.951**	-0.056	-0.010	-0.086	-0.068
Errors median reaction time	**0.462**	-0.075	-0.139	-0.364	-0.046	-0.038	0.105
Stroop test							
Total time	**0.925**	0.016	0.094	-0.044	0.060	0.055	-0.030
Median reaction time in 1st task	**0.379**	-0.139	0.269	-0.353	0.427	0.423	-0.062
Median reaction time in 2st task	**0.854**	-0.031	0.083	-0.121	0.125	0.123	-0.062
Median reaction time in 3st task	**0.878**	0.121	0.039	0.075	0.089	-0.141	-0.064
Interference	**0.783**	0.196	-0.090	0.278	-0.134	-0.400	-0.037
Simple and choice reaction time tests							
Median simple reaction time	-0.031	0.032	0.142	**-0.911**	0.019	-0.008	0.044
Total simple reaction time	0.035	0.085	0.130	**-0.912**	-0.018	-0.016	0.064
Median choice reaction time	0.404	0.334	0.028	**-0.536**	0.034	-0.022	0.053
Total choice reaction time	0.456	0.306	0.045	**-0.502**	0.019	-0.009	0.060
Implicit learning test							
Implicit learning	-0.118	0.061	-0.044	-0.103	0.141	-0.064	**0.913**
Implicit learning interference	0.179	-0.034	0.218	0.522	-0.101	0.487	**0.319**
Visual and spatial short-term memory test							
Reaction time in direct order	0.006	0.116	-0.064	-0.043	**0.856**	-0.073	-0.029
Reaction time in direct order	-0.042	0.037	-0.067	0.152	**0.946**	-0.048	0.149
Survey test							
Median reaction time 1st task	0.222	-0.065	0.110	-0.111	0.151	**-0.745**	0.087
Median reaction time 2nd task	0.117	-0.153	0.301	-0.074	0.184	**-0.740**	0.054
Median reaction time 3rd task	0.168	-0.078	0.555	0.135	0.038	**-0.509**	0.076

Values in bold indicate the variables included in each component.

**Table 7.** Phi matrix.

	1	2	3	4	5	6	7
1	1.000	0.223	0.251	-0.268	0.235	-0.235	0.042
2		1.000	0.176	-0.181	0.262	-0.029	0.018
3			1.000	-0.108	0.201	-0.059	0.024
4				1.000	-0.251	0.040	0.024
5					1.000	-0.146	0.015
6						1.000	0.019
7							1.000

## DISCUSSION

Findings suggest that CompCog is valid for measuring the cognitive processes its tasks intend to evaluate. Regarding PCA’s results, the items of each subtest were loaded on the same component. This suggests that each component represents one test that do not overlap, except for Simple Reaction Time and Choice Reaction Time, which are loaded together. The correlations between components’ scores, as shown in Table 7, were also low. Three variables deserve further discussion: Error’s median reaction time of Inhibitory control test; Median reaction time in 1st task of the Stroop Test; and Implicit learning interference of Implicit learning test.

The latter could be placed in different components, with the possibility of being included together with the other variable of the Implicit learning test. Other options are in the Survey component and Stroop component. Actually, the variable is a control measure for the Implicit learning test (Table 1) and uses some of the same cognitive functions of the components where it loaded. The same can be said about the Median reaction time in 1st task of the Stroop Test. The variable could be placed in three components, including altogether with the other variables of the same test. The 1st task of the Stroop Test does not involve the Stroop effect yet, and it is a measure of attention and used also to calculate the interference. The last variable worth discussing is the Error’s median reaction time of Inhibitory control test. It loaded together with Stroop Test’s variables. One possibility to explain this item loading component is that errors might create a different pattern when compared to correct answers — the majority of measures in this study. Error’s reaction time and what this type of measure can tell us might be something worth exploring in future studies.

Also, the results have shown moderate correlations (0.30<r<0.49) and strong correlations (r>0.5) (28) between CompCog’s tasks and their equivalents considering paper and pencil tests. Some factors can explain the small number of strong correlations. First, it is important to highlight the differences between both assessments used. Besides all the particularities that computerized tests have in relation to paper and pencil tasks, CompCog is highly dependent on visual and motor stimuli, something unusual when considering traditional cognitive tasks that rely on paper, pencil, and oral interactions. Other studies that aimed to produce concurrent validity evidence of computerized tests comparing it to traditional analogous tests had the same results.^29,30^ Second, the target group to which CompCog was designed, i.e., elderly and AD patients,^18^ is not the same group that composed the study sample. This brought some facilities to the study realization and comparisons, such as higher subjects’ availability and normal cognitive performance in both batteries, but it also created a ceiling effect in some tasks that may have influenced the analyses. The high diagnostic accuracy reported in other studies is, itself, another evidence of the validity of CompCog’s tasks.^18,27^

### Simple reaction time and choice reaction time

Both tests assess processing speed and attention, and its variables were correlated to TEACO-FF, TECON 1, and WAIS Processing Speed Index. The correlations are negative because CompCog measures are provided in time, while the analogous tests have the variables of correct answers. Choice Reaction Time seems to be a better measure of processing speed than the Simple Reaction Time. An explanation is a possibility of creating an automatic pattern of hitting the screen in the Simple Reaction Time test, something that cannot be performed in the Choice Reaction Time as it involves a choice between options.

### Implicit learning test

There are not many traditional paper and pencil tests that specifically measure implicit learning, possibly due to the difficulty of assessing this cognitive function through paper and pencil tests. Nevertheless, WCST and R-1 Test are the options that, even though not primarily, involve implicit processes.^31,32^ CompCog’s implicit learning variable was correlated with R-1 score (r=-0.305, p=0.031). R-1 involves insight and implicit perception,^31^ as the implicit learning variable also does. The correlation is negative because the smaller the variable is, the higher the implicit learning. The implicit learning interference measured through CompCog had a significant negative correlation with WCST variable trials to complete first category (r=-0.410, p=0.003). This can be explained as the process to learn the rule to complete the first category in WCST involves implicit learning.^32^ The more trials one needs to complete the category in WCST, the less implicit learning happened in the CompCog task, the smaller the interference of this learning when completing the last sequence. The other variables suffer less influence of implicit learning, being this probably the explanation for the absence of other correlations.

### Visual and spatial short-term memory

There were almost no correlations between CompCog’s short-memory task and short-memory traditional paper and pencil tests. However, it is important to notice that the CompCog’s task is based on the visual and spatial stimuli, while WAIS-III variables are based on the auditory stimuli. It is widely accepted that, although there is a common component for both stimuli processing in working memory (i.e., central executive),^33^ there are different processing pathways for each one of them. Auditory stimuli should be processed in the Phonological Loop, and the visual and spatial stimuli, in the Visuospatial Sketchpad,^33^ making both tests challenging to compare.

### Face recognition and memory

The tasks of Rey Auditory Verbal Learning Test (RAVLT) and CompCog had moderate correlations regarding only the first CompCog’s task and the correct answers’ total percentage. An explanation for the lack of correlations between the other tasks and RAVLT variables is the ceiling effect seen on the computerized test. As it was developed considering the memory capacity of elderly and AD patients, university students could quickly learn the stimuli presented in the first try, creating an almost constant variable on the subsequent tasks. Besides it, RAVLT involves auditory stimuli while CompCog considers the memory of visual stimuli. Rey Figure Test, instead, assesses visual memory, but it is a test that suffers a lot of influence of executive functions, ^34^ which does not happen in CompCog. Even so, the moderated correlations were found between the recall score of Rey Figure Test and the median reaction time of the computerized test’s 1st and 2nd tasks. Again, the ceiling effect may have contributed to the lack of correlations regarding the other two tasks.

### Inhibitory control test

Inhibitory control is an executive function that requires the inhibition of automatic processes in order to activate controlled processes. This cognitive function is evaluated in Stroop’s 3rd task and Color Trails test, with which CompCog’s Inhibitory Control Test had moderated correlations. Total time, median reaction time, and correct answer’s median reaction time were correlated with Stroop’s 3rd task. This makes sense since the last task needs more time to be completed if they take a longer time to produce their answers due to slower processing related to inhibitory control. Instead, Error’s median reaction time was correlated with Color Trails’ 2nd task. A more significant Error’s median reaction time shows a situation where inhibitory control actually did not work out. The same can be said about time to completion in Color Trails’ 2nd task.

### Stroop test

Stroop’s paper and pencil test and its computerized version are both based on the same paradigm. Even so, there are still some differences between both versions. Correlations were found between the 2nd task of each presentation, 3rd task, and interference. An explanation for the lack of correlation between the 1st tasks — the only one not correlated — is that the paper and pencil version is more an automatic process — naming colors — than the computerized version. The latter involves the time to choose between the colors’ buttons at the bottom of the screen, which is less automatic than naming.

### Survey test

Survey is a component of attention and was mainly correlated to TEACO-FF, the traditional test most related to the CompCog’s survey test. Both involve the survey for a simple stimulus (one figure or colors). The correlation is negative because the CompCog’s test is measured through time — the smaller, the better — and the paper and pencil test is measured through correct answers — the higher, the better. TECON 1 did not present correlations with the computerized test, possibly due to the higher complexity of the stimulus that has to be searched, what demands working memory, and divided attention besides survey capacity.

A limitation of this study is, as already mentioned, the differences between the characteristics of the sample used in this study and the population to which CompCog was created to.^18^ Nevertheless, the chosen sample brought benefits, too, such as preserved cognitive abilities to assess and compare the processes underneath each neuropsychological test. The differences between both assessments — computerized and paper and pencil — can also be considered as a limitation, but, at the same time, it is the option available to assess the concurrent validity of new computerized neuropsychological batteries. Nevertheless, the high number of measures based on reaction time and total time is an important advantage of CompCog and other computerized tests. The precise measurement given by time variables, with high sensibility and results with millisecond precision, is even more accurate than correct answers to evaluate cognitive processes,^19^ as it may assess subtle changes in cognition that might not have impacted the outcome yet. Moreover, the PCA supported the differentiation between cognitive domains measured by the subtests of the battery. In conclusion, the results suggest that CompCog is a valid measure of memory, attention, implicit learning, inhibitory control, and processing speed.
